# The performance of ASpirin-FREE therapy after successful percutaneous coronary intervention for acute coronary syndrome: the ASFREE prospective pilot study

**DOI:** 10.12701/jyms.2026.43.25

**Published:** 2026-03-19

**Authors:** Donghyeon Joo, Sungho Jo, Jeong Tae Byoun, Jae Young Cho, Kyeong Ho Yun

**Affiliations:** Department of Cardiovascular Medicine, Regional Cardiocerebrovascular Center, Wonkwang University Hospital, Iksan, Korea

**Keywords:** Aspirin, P2Y12 inhibitors, Percutaneous coronary intervention, Treatment outcome

## Abstract

**Background:**

Dual antiplatelet therapy with aspirin and a P2Y12 inhibitor is standard after percutaneous coronary intervention (PCI) for acute coronary syndrome (ACS); however, bleeding risk remains a major concern. Early discontinuation of aspirin due to potent P2Y12 inhibition may mitigate bleeding without increasing thrombotic events.

**Methods:**

The ASpirin-FREE therapy after successful percutaneous coronary intervention for acute coronary syndrome (ASFREE) study was an investigator-initiated, single-center, prospective, open-label, single-arm pilot study enrolling patients with ACS who underwent PCI with drug-eluting stents. All patients received a single loading dose of aspirin on the day of the PCI, followed by ticagrelor or prasugrel monotherapy. The primary efficacy endpoint was target vessel failure (TVF) at 12 months. The primary safety endpoint was definite stent thrombosis. Event rates are reported with 95% confidence intervals (CIs).

**Results:**

In total, 228 patients were enrolled. TVF occurred in 10 patients (4.4%; 95% CI, 2.1%–7.9%). Definite stent thrombosis was observed in one patient (0.4%; 95% CI, 0.01%–2.4%), with no acute or subacute events. Major bleeding (Bleeding Academic Research Consortium type 3 or 5) occurred in two patients (0.9%; 95% CI, 0.1%–3.1%).

**Conclusion:**

An aspirin-free strategy following a single loading dose with continuation of potent P2Y12 inhibitor monotherapy was feasible in patients with ACS undergoing PCI and was associated with low rates of thrombotic and major bleeding events. These findings should be regarded as hypothesis-generating and supporting further evaluations in adequately powered randomized controlled trials (CRIS registration: KCT0008182).

## Introduction

Dual antiplatelet therapy (DAPT) with aspirin and P2Y12 inhibitors remains the cornerstone strategy for preventing thrombosis in patients who are at high risk with coronary artery disease, particularly those with acute coronary syndrome (ACS) undergoing percutaneous coronary intervention (PCI) [1,2]. Although this combination has proven effective in reducing thrombotic complications, it is associated with an elevated risk of bleeding, thereby necessitating the investigation of alternative strategies to alleviate bleeding risk without compromising antithrombotic efficacy [3]. Studies have suggested that a shortened course of DAPT (e.g., 1–3 months) followed by P2Y12 inhibitor monotherapy can effectively reduce major bleeding events while maintaining comparable protection against ischemic outcomes [4-9].

The Acetyl Salicylic Elimination Trial (ASET) demonstrated that aspirin-free prasugrel monotherapy after stent implantation was a safe strategy without stent thrombosis during the 3-month follow-up period in patients with stable coronary artery disease [10]. However, this strategy has not been evaluated in patients with ACS. This study aims to evaluate the efficacy and safety of potent P2Y12 inhibitor monotherapy, following aspirin discontinuation after a single loading dose, in patients with ACS who underwent PCI with drug-eluting stents (DES).

## Methods

**Ethics statement:** This study protocol was reviewed and approved by the Institutional Review Board including the Ethics Committee of Wonkwang University Hospital (IRB No: 2022-10-022). Written informed consent was obtained from all participants prior to study enrollment.

### 1. Study design

The ASpirin-FREE therapy after successful percutaneous coronary intervention for acute coronary syndrome (ASFREE) study is an investigator-initiated, single-center, prospective, open-label, single-arm pilot study (CRIS registration: KCT0008182). Considering the exploratory nature of this study, formal sample size calculations were not performed. Instead, a precision- and safety-oriented approach was adopted, consistent with previous first-in-human or feasibility studies that evaluated novel antiplatelet strategies. The planned enrollment of approximately 200 patients was considered sufficient to provide an initial estimate of event rates with a reasonable confidence interval (CI) width, while limiting patient exposure in the absence of prior data in an ACS population. Patient enrollment was discontinued if two or more cases of definite stent thrombosis occurred during the 1-year follow-up period. The above figure was based on the incidence of stent thrombosis (approximately 0.7%) reported in the Ticagrelor Monotherapy after 3 Months in the Patients Treated with New Generation Sirolimus Eluting Stent for Acute Coronary Syndrome (TICO) trial and a large-scale Korean myocardial infarction registry [8,11]. Therefore, the stopping rule was designed to identify an early and clinically unacceptable excess risk rather than to formally test noninferiority. Accordingly, the primary objective of the present study was to assess feasibility and generate safety signals, rather than to establish comparative efficacy.

Patients requiring PCI with DES for ST-segment elevation myocardial infarction (MI) or those with non-ST-segment elevation ACS were eligible for the study. Patients were excluded from the study if they met any of the following criteria: (1) age <19 years, (2) known intolerance to prasugrel or ticagrelor, (3) history of intracranial hemorrhage, (4) active bleeding, (5) life expectancy of <1 year, and (6) any condition deemed inappropriate for study participation at the discretion of the investigator.

### 2. Study procedures

PCI with DES implantation was performed in accordance with established clinical practice guidelines. PCI success was defined as achievement of thrombolysis in myocardial infarction (TIMI) flow grade ≥2 and acceptable residual stenosis (<30%) based on angiographic assessment. All enrolled patients received a loading dose of aspirin (300 mg) combined with either ticagrelor (180 mg) or prasugrel (60 mg). Maintenance antiplatelet therapy was initiated with prasugrel 10 mg once daily or ticagrelor 90 mg twice daily, and aspirin was discontinued immediately after PCI. The P2Y12 inhibitor was selected at the discretion of the treating physician. It was discontinued in patients who had been pretreated with clopidogrel, and at the investigator’s discretion, a loading dose of prasugrel (40 mg) or ticagrelor (180 mg) was administered on the day of the procedure or the following day, followed by initiation of maintenance therapy. The concomitant use of other antiplatelet agents or anticoagulants was not permitted.

All study patients underwent platelet function testing before discharge using the VerifyNow P2Y12 assay (Werfen, Barcelona, Spain). The platelet reactivity >208 P2Y12 reaction units (PRUs) was defined as high platelet reactivity (HPR) [12].

Clinical follow-up was performed 1 month and 1 year post-procedure. Patient symptoms, treatment adherence, and clinical outcomes were evaluated by reviewing medical records. In cases where in-person visits were not feasible, follow-ups were conducted via telephone interviews.

### 3. Study endpoints

The primary efficacy endpoint was the incidence of target vessel failure (TVF) at 12 months, which was analyzed in the intention-to-treat population. TVF was defined as a composite of cardiac death, target vessel-dependent MI, and target vessel revascularization [13]. The primary safety endpoint was the incidence of definite or probable stent thrombosis. Secondary endpoints included major adverse cardiac events (MACE), defined as a composite of all-cause mortality, fatal or nonfatal MI, ischemic stroke, and any revascularization. Additional secondary outcomes included the incidence of bleeding events based on the Bleeding Academic Research Consortium (BARC) definition, specifically examining BARC types 2, 3, and 5 bleeding events [14]. Major bleeding was considered BARC type 3 or 5 bleeding.

### 4. Statistical analysis

All analyses were conducted in accordance with the intention-to-treat principle. Continuous variables are presented as mean±standard deviation or as median with interquartile range (IQR), as appropriate. Event rates are reported as counts and percentages. Two-sided 95% CIs for proportions were calculated using the exact Clopper-Pearson method based on a binomial distribution given the relatively low number of events. For censored cases, the last available data point was used for the survival analysis. Comparisons with previously published trials were descriptive and based on the historical event rates. No formal statistical comparisons were made between this single-arm cohort and external control groups. All statistical analyses were performed using IBM SPSS ver. 29.0 (IBM Corp., Armonk, NY, USA).

## Results

### 1. Patient characteristics

**Fig. 1.**
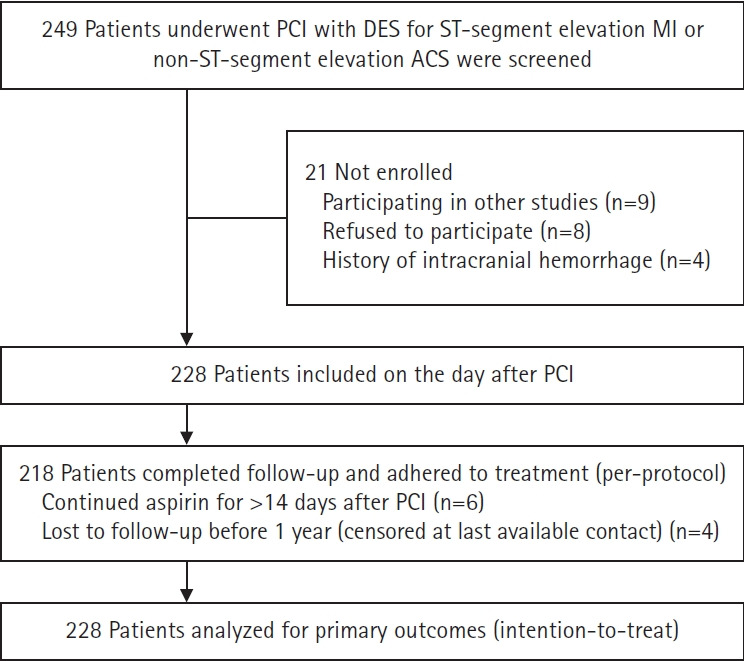
Participant flow. Patients lost to follow-up before 1 year are censored at the time of last contact in the time-to-event analyses. All patients are analyzed according to the intention-to-treat principle. ACS, acute coronary syndrome; DES, drug-eluting stent(s); MI, myocardial infarction; PCI, percutaneous coronary intervention.

Between October 2022 and October 2023, 228 patients with ST-segment elevation MI or non-ST-segment elevation ACS who underwent PCI with DES were enrolled. The flow diagram of this study is shown in Fig. 1. The clinical and angiographic characteristics of the study population are summarized in Table 1. The mean age was 61.2±9.2 years, and 86.4% of the patients were male. Multivessel coronary artery disease was present in approximately 40% of the patients. The procedural details are provided in Table 2. Radial artery access was used for PCI in 50.9% of patients, and sirolimus-eluting stents were implanted in 38.6% of the patients. A post-procedural TIMI flow grade of 3 was achieved in 96.9% of the treated lesions.

Adherence to the study medications is summarized in Table 3. Eleven patients who were receiving chronic aspirin therapy continued to receive a maintenance dose of aspirin (100 mg) before the procedure. The per-protocol population was 218 (97.4%), and six patients did not discontinue aspirin within 14 days after PCI (aspirin cover date, 1.9 days; IQR, 1.0–2.0 days). At the 1-year follow-up, all 228 intent-to-treat patients (100%) completed the clinical evaluation and were analyzed. Among the two patients who initially received a clopidogrel loading dose, one was switched to prasugrel and the other to ticagrelor. In one additional patient who had been maintained on clopidogrel without a loading dose, the therapy was switched to prasugrel at the investigator’s discretion.

### 2. Clinical outcomes

**Fig. 2.**
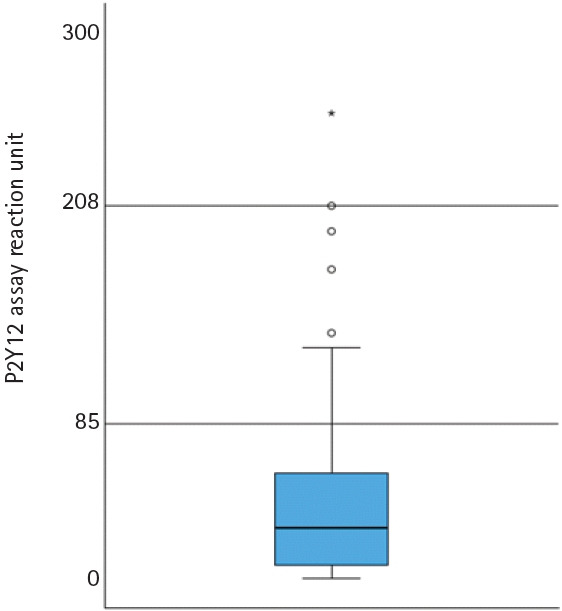
Platelet reactivity before discharge. The median P2Y12 reaction unit value is 28 (interquartile range, 7.0–58.5).

At discharge, the median PRU value was 28 (IQR, 7.0–58.5), with only one patient (0.4%) demonstrating HPR (Fig. 2). Given the limited number of clinical events, no formal analysis of the relationship between PRU levels and patient outcomes was performed.

**Fig. 3.**
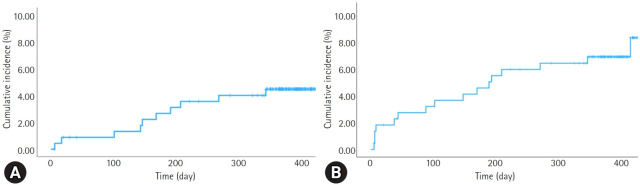
Kaplan-Meier curves for (A) target vessel failure (TVF) and (B) major adverse cardiac events (MACE). At 1 year, TVF occurred in 10 patients (4.4%) and MACE occurred in 16 patients (7.0%).

The clinical outcomes at 1-year follow-up are summarized in Table 4 and Fig. 3. The primary efficacy endpoint occurred in 10 patients (4.4%; 95% CI, 2.1%–7.9%) and included four cardiac deaths, two target vessel-dependent MIs, and seven target vessel revascularizations. Definite stent thrombosis was observed in one patient (0.4%; 95% CI, 0.01%–2.4%) at day 187. No acute or subacute stent thrombosis events were observed. Secondary outcomes included MACE in 16 patients (7.0%; 95% CI, 4.1%–11.1%), all-cause mortality in five patients (2.2%), MI in three patients (1.3%), ischemic stroke in four patients (1.8%), and any revascularization in 10 patients (4.4%). Bleeding events were reported in 13 patients, with major bleeding (BARC type 3 or 5) occurring in only two patients (0.9%; 95% CI, 0.1%–3.1%).

The baseline characteristics, platelet reactivity, and 1-year clinical outcomes stratified by P2Y12 inhibitor (ticagrelor vs. prasugrel) are presented in Table 5. These data are descriptive in nature, as the study was not powered to support formal comparisons between treatment groups.

## Discussion

This study evaluated the feasibility of an aspirin-free antiplatelet strategy using ticagrelor or prasugrel monotherapy after PCI in patients with ACS. The key findings were as follows: (1) aspirin discontinuation with continued ticagrelor or prasugrel use was associated with an acceptable 1-year TVF rate of 4.4%, (2) major bleeding was infrequent, occurring in 0.9% of patients, and (3) definite or probable stent thrombosis occurred in 0.4% of patients. In a Korean nationwide registry, the incidence of major bleeding events in patients with ACS receiving DAPT was 7.3% with ticagrelor and 7.9% with prasugrel, whereas the incidence of ischemic events, defined as a composite of death, MI, and stroke, was 5.6% with ticagrelor and 6.1% with prasugrel [15]. In this context, the low rates of ischemic and bleeding events observed in the present study suggest that an aspirin-free strategy with potent P2Y12 inhibition is feasible and provides a favorable balance between ischemic protection and bleeding risk. However, these observations should be interpreted cautiously given the single-arm design and should be regarded as hypothesis-generating rather than indicative of comparative efficacy.

Current clinical guidelines recommend 12 months of DAPT for patients with ACS [1,2]. However, concerns regarding the heightened bleeding risk remain and the optimal timing and choice of agent for de-escalation to single antiplatelet therapy remain unclear. Several randomized clinical trials have evaluated the strategy of P2Y12 inhibitor monotherapy following short-term DAPT [4-9]. Among these, the TICO and Short and Optimal Duration of Dual Antiplatelet Therapy After Everolimus-Eluting Cobalt-Chromium Stent-2 (STOPDAPT-2) ACS trials are the only randomized studies conducted specifically in patients with ACS [8,9]. In the TICO trial, the first randomized controlled trial conducted exclusively in patients with ACS, aspirin was discontinued at 3 months post-PCI, and patients continued ticagrelor monotherapy thereafter. The trial demonstrated a notable decrease in the combined outcome of major bleeding and cardiovascular events at the 1-year follow-up [8]. Conversely, the STOPDAPT-2 ACS trial demonstrated that clopidogrel monotherapy following 1 to 2 months of DAPT after PCI did not confer a net clinical benefit as the decline in bleeding was offset by an increase in cardiovascular events [9]. These discrepant findings highlight the importance of using potent P2Y12 inhibitors in abbreviated DAPT strategies for ACS. Our study supports the importance of potent P2Y12 inhibition in ACS and indicates that the duration of aspirin exposure after PCI may be minimized. Moreover, the recent Ticagrelor Monotherapy in Patients Treated with New-Generation Drug-Eluting Stents for Acute Coronary Syndrome (T-PASS) trial further reinforced this concept, demonstrating that aspirin can be safely discontinued within 1 month (median, 16 days) while maintaining ticagrelor monotherapy.

The adenosine diphosphate (ADP)-induced pathway plays a pivotal role in thrombus formation. In ACS or PCI, platelet activation triggers the release of ADP, which, in turn, stimulates P2Y12 receptors on neighboring platelets and promotes further aggregation. Therefore, inhibition of the ADP-induced pathway is a fundamental principle of antiplatelet therapy in patients undergoing stent implantation [16]. This concept is corroborated by findings from the Assessment of Dual Antiplatelet Therapy with Drug-Eluting Stents (ADAPT-DES) study, which showed that high on-treatment platelet reactivity to ADP following clopidogrel administration was significantly associated with an increased risk of stent thrombosis within 1 year (hazard ratio, 2.49; *p*=0.001). In contrast, HPR to arachidonic acid, which reflects aspirin-related pathways, was not significantly associated with stent thrombosis [17]. These findings underscore the critical role of suppressing the ADP-induced pathway in preventing thrombotic complications, including stent thrombosis and suggest that pathways such as the arachidonic acid-induced pathway play a more supportive role. Moreover, evidence from other studies suggests that effective inhibition of the ADP-induced pathway indirectly suppresses the arachidonic acid-induced pathway, indicating that the incremental benefit of aspirin may be minimal when potent P2Y12 inhibitors are used [18,19]. In the Clopidogrel Versus Aspirin in Patients at Risk of Ischemic Events (CAPRIE) trial, a pooled analysis of patients with prior stroke, MI, or peripheral arterial disease demonstrated that clopidogrel was associated with an 8.7% relative risk reduction in composite ischemic events compared with aspirin [20]. Additionally, the Harmonizing Optimal Strategy for Treatment of Coronary Artery Stenosis–Extended Antiplatelet Monotherapy (HOST-EXAM) trial found that clopidogrel reduced thrombotic events by 32% over a 2-year follow-up compared with aspirin among patients who had undergone stent implantation 6 to 18 months earlier [21]. These findings suggest that inhibition of the ADP pathway may offer broader clinical protection than blocking the thromboxane A2 pathway.

The thrombotic risk following ACS is the highest during the initial 3 months [22]. In the T-PASS trial, early discontinuation of aspirin at a median of 16 days (IQR, 12–25 days) in patients with ACS treated with ticagrelor and new-generation DES did not increase the ischemic risk compared with continued DAPT [23]. These findings indicate that aspirin may only be necessary during the immediate post-PCI period. In the present study, the 1-year rate of definite stent thrombosis was low (0.4%) with an upper 95% confidence bound of 2.4%. Although no formal noninferiority testing was performed due to the single-arm design, the upper bound of the 95% CI for definite stent thrombosis remained within the range of event rates reported in contemporary ACS trials using standard DAPT, including the TICO trial [8]. Importantly, no acute or subacute stent thrombosis events were observed, and only a single event occurred in the late phase. However, these observations should be viewed as hypothesis-generating rather than confirmatory, and definitive conclusions regarding comparative safety require validation in adequately powered randomized controlled trials.

In addition to the ASET study, the Optical Coherence Tomography-Guided PCI with Single-Antiplatelet Therapy (OPTICA) trial recently demonstrated the feasibility of initiating ticagrelor or prasugrel monotherapy immediately after PCI in patients with non-ST-segment elevation ACS [24]. Our findings extend this strategy to a broader ACS population, including patients with ST-segment elevation MI. Given the limited incremental antiplatelet effects of aspirin, the potent P2Y12 inhibition by ticagrelor or prasugrel supports the rationale for early aspirin discontinuation. In our cohort, approximately 90% of the patients exhibited high levels of platelet inhibition (<85 PRUs) with infrequent major bleeding events, suggesting that early aspirin withdrawal reduces bleeding risk without compromising ischemic protection.

This study has certain limitations. First, the single-arm nonrandomized design inherently limits causal inference and precludes direct comparison with standard DAPT strategies. As such, the findings should be interpreted as exploratory and hypothesis-generating rather than confirmatory. Second, the relatively small sample size and low event rates restrict the statistical power to detect infrequent safety outcomes, particularly definite stent thrombosis. Although event rates were reported with exact CIs, the upper bounds of these intervals remained wide, underscoring the need for cautious interpretation. Third, the selection of P2Y12 inhibitor (ticagrelor or prasugrel) was left to the physician’s discretion rather than randomized allocation, introducing potential confounding and treatment selection biases that could not be fully accounted for in this study. Fourth, because this was a single-center study conducted in a specific clinical setting with contemporary PCI techniques and high adherence to potent P2Y12 inhibitors, the generalizability of the findings to other populations or practice environments may be limited. Finally, although platelet reactivity was systematically assessed, the limited number of clinical events precluded a robust evaluation of the relationship between on-treatment platelet reactivity and clinical outcomes. Taken together, these limitations highlight the need for adequately powered randomized controlled trials to definitively evaluate the safety and efficacy of aspirin-free strategies in patients with ACS undergoing PCI.

In conclusion, in patients with ACS, an aspirin-free strategy, initiated after a single loading dose and followed by ticagrelor or prasugrel monotherapy post-PCI, was shown to be both feasible and safe. These findings provide preliminary evidence supporting the potential role of potent P2Y12 inhibitor monotherapy and warrant further investigation in adequately powered randomized controlled trials comparing this approach with standard DAPT in contemporary PCI settings.

## Figures and Tables

**Table 1.** Clinical and angiographic characteristics

Characteristic	Value
No. of patients	228
Age (yr)	61.2±9.2
Male sex	197 (86.4)
Hypertension	129 (56.6)
Diabetes mellitus	86 (37.7)
Current smoking	102 (44.7)
Previous myocardial infarction	15 (6.6)
Previous percutaneous coronary intervention	26 (11.4)
Clinical presentation	
ST-segment elevation MI	81 (35.5)
Non–ST-segment elevation MI	51 (22.4)
Unstable angina	93 (40.8)
Silent ischemia	3 (1.3)
LVEF on echocardiography (%)	53.7±9.7
Coronary artery disease	
1-Vessel	137 (60.1)
2-Vessel	68 (29.8)
3-Vessel	23 (10.1)
Culprit lesion location	
Left main artery	9 (3.9)
Left anterior descending artery	115 (50.4)
Left circumflex artery	38 (16.7)
Right coronary artery	66 (28.9)

Values are presented as number only, mean±standard deviation, or number (%). MI, myocardial infarction; LVEF, left ventricular ejection fraction.

**Table 2.** Procedural characteristics (n=228)

Characteristic	Value
Preprocedural TIMI flow grade	
0/1	108 (47.4)
2/3	120 (52.6)
Radial access	116 (50.9)
Total stent length (mm)	42.2±29.1
Number of lesions treated	1.4±0.7
Number of stents implanted	1.6±0.9
Stent type	
Sirolimus-eluting	88 (38.6)
Biolimus-eluting	75 (32.9)
Everolimus-eluting	61 (26.8)
Novolimus-eluting	4 (1.8)
Postdilatation with noncompliant balloon	85 (37.3)
Intravascular ultrasound use	40 (17.5)
Optical coherence tomography use	4 (1.8)
Postprocedural TIMI flow grade	
0/1	2 (0.9)
2/3	226 (99.1)
Complex percutaneous coronary intervention	
≥3 lesions treated	16 (7.0)
≥3 stents implanted	25 (11.0)
Total stent length >60 mm	43 (18.9)
Bifurcation with 2 stents implanted	3 (1.3)
Chronic total occlusion	7 (3.1)

Values are presented as number (%) or mean±standard deviation. TIMI, thrombolysis in myocardial infarction.

**Table 3.** Adherence to study medications

Variable	Value
Initial loading	
P2Y12 inhibitors	228 (100)
Ticagrelor	164 (71.9)
Prasugrel	61 (26.8)
Clopidogrel	2 (0.9)
None	1 (0.4)
Aspirin	217 (95.2)
Duration of aspirin use (day)	1.9±2.5
Discharge	
P2Y12 inhibitors	228 (100)
Ticagrelor	165 (72.4)
Prasugrel	63 (27.6)
Clopidogrel	0 (0)
Aspirin	0 (0)

Values are presented as number (%) or mean±standard deviation.

**Table 4.** Clinical outcomes at 1 year

Variable	Value	95% CI
Stent thrombosis	1 (0.4)	0.01–2.4
Target vessel failure	10 (4.4)	2.1–7.9
Cardiac death	4 (1.8)	0.5–4.4
Target vessel myocardial infarction	2 (0.9)	0.1–3.1
Target vessel revascularization	7 (3.1)	1.3–6.3
Major adverse cardiac events	16 (7.0)	4.1–11.1
All-cause death	5 (2.2)	0.7–5.0
All myocardial infarction	3 (1.3)	0.3–3.8
Any revascularization	10 (4.4)	2.1–7.9
Ischemic stroke	4 (1.8)	0.5–4.4
Bleeding events	13 (5.7)	3.1–9.4
BARC type 2	11 (4.8)	2.4–8.5
BARC type 3a	1 (0.4)	0.01–2.4
BARC type 3b	1 (0.4)	0.01–2.4
BARC type 3c	0 (0)	
BARC type 5	0 (0)	
Major bleeding (BARC 3 or 5)	2 (0.9)	0.1–3.1

Values are presented as number (%) unless otherwise specified. CI, confidence interval; BARC, Bleeding Academic Research Consortium.

**Table 5.** Baseline characteristics and clinical outcomes according to type of P2Y12 inhibitor

Variable	Ticagrelor (n=165)	Prasugrel (n=63)
Age (yr)	62.2±9.4	58.6±8.2
Male	137 (83.0)	60 (95.2)
Hypertension	104 (63.0)	25 (39.7)
Diabetes mellitus	64 (38.8)	22 (34.9)
Current smoker	65 (39.4)	37 (58.7)
Myocardial infarction (index event)	95 (57.6)	37 (58.7)
Complex percutaneous coronary intervention	40 (24.2)	6 (9.5)
P2Y12 reaction unit	28 (7–57)	27 (5–62)
<85	151 (91.5)	55 (87.3)
85–208	13 (7.9)	8 (12.7)
>208	1 (0.6)	0 (0)
Stent thrombosis	1 (0.6)	0 (0)
Target vessel failure	8 (4.8)	2 (3.2)
Major adverse cardiac events	12 (7.3)	4 (6.3)
Major bleeding (BARC 3 or 5)	2 (1.2)	0 (0)

Values are presented as mean±standard deviation, number (%), or median (interquartile range). PCI, percutaneous coronary intervention; BARC, Bleeding Academic Research Consortium. Complex PCI was defined as the presence of at least one of the following: ≥3 lesions treated, ≥3 stents implanted, total stent length >60 mm, bifurcation with 2 stents implanted, or chronic total occlusion intervention. Major adverse cardiac events were defined as a composite of all-cause death, myocardial infarction, ischemic stroke, and revascularization.
